# Case Report: An Undefined Liver Lesion in a Young Man With Severe Aplastic Anemia: A Teachable Moment

**DOI:** 10.3389/fsurg.2021.665367

**Published:** 2021-07-14

**Authors:** Jin Liu, Jidong Sui, Deguang Sun, Kun Guo, Zhenming Gao, Jie Bian, Jinsong Yan, Liming Wang

**Affiliations:** ^1^Division of Hepatobiliary and Pancreatic Surgery, Department of General Surgery, The Second Affiliated Hospital of Dalian Medical University, Dalian, China; ^2^Department of Pathology, The Second Affiliated Hospital of Dalian Medical University, Dalian, China; ^3^Department of Radiology, The Second Affiliated Hospital of Dalian Medical University, Dalian, China; ^4^Department of Hematopathology, The Second Affiliated Hospital of Dalian Medical University, Dalian, China

**Keywords:** androgen-related hepatic adenoma, extramedullary hematopoiesis, a teachable moment, less is more, clinical challenges

## Abstract

In this work, we reported a young man complaining of asthenia and intermittent fever for 10 days, and an ultrasound showed an undefined lesion on his liver. Facing the patient's situation with severe agranulocytosis, anemia, and thrombocytopenia, we passed through a tough diagnostic process for choosing an appropriate treatment for him with an ambiguous result of pathological biopsy. The undefined liver lesion was successfully solved by withdrawing the androgen for observation, without lobectomy. The lesion gradually diminished over 2 years of follow-up.

## Introduction

Androgen-related hepatic adenoma happens occasionally in people who take androgen for therapy (1), such as haematopoietic dysfunction, hypogonadism, osteoporosis, endogenously elevated titres of androgens like polycystic ovary syndrome, even some athletes, which confound doctors' diagnoses, especially oncological surgeons. It is hoped that this case report will prevail upon the surgeons the importance of taking every hint of diseases and the tenet of “Less is more.”

## Case Presentation

**Figure 1:**
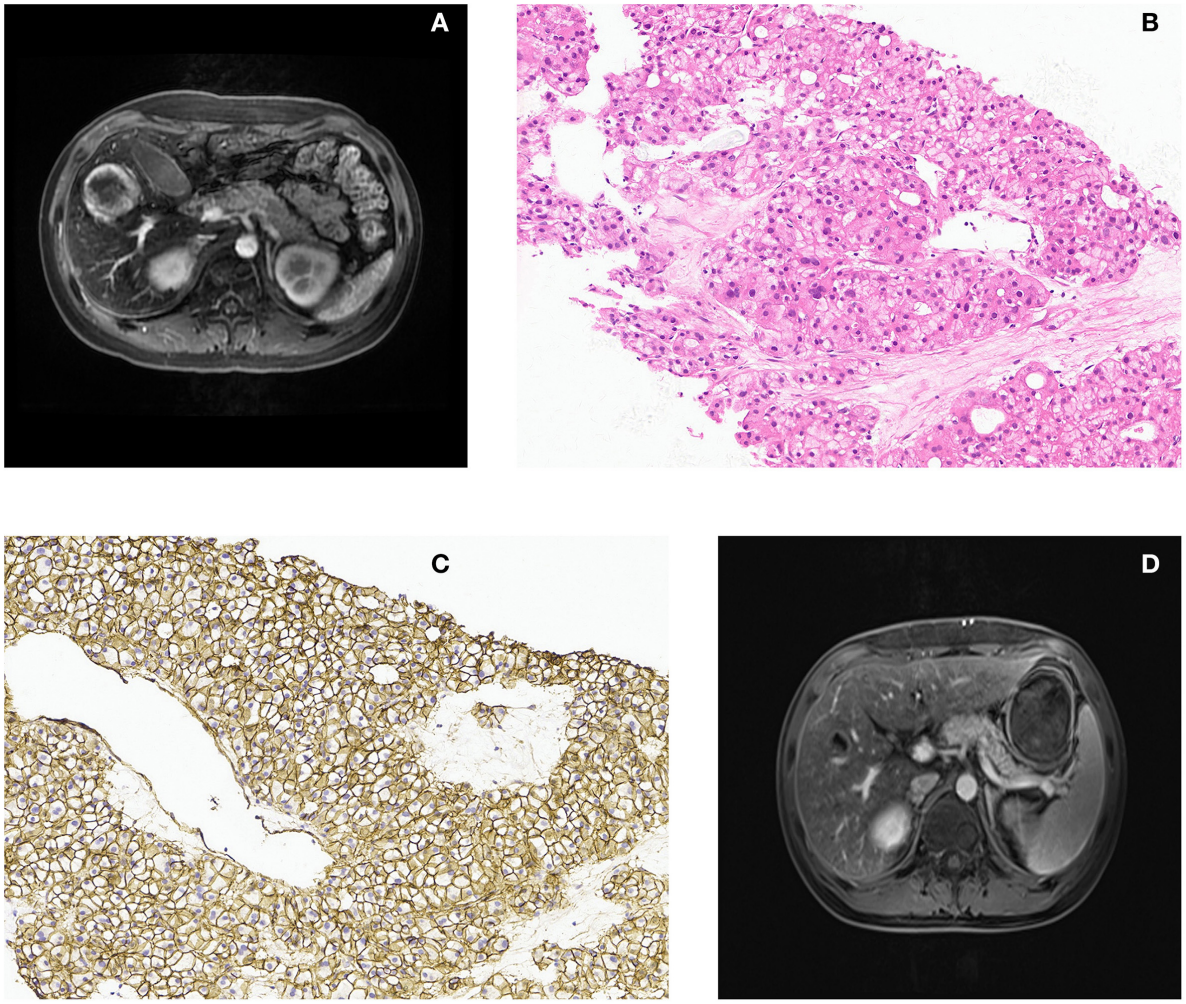
Magnetic resonance imaging characteristics and liver biopsy histopathology. **(A)** Axial view from enhanced abdominal magnetic resonance imaging revealed a liver mass located in the right lobe without typical enhancement. **(B)** Hematein eosin stain composed of a small amount of abnormal mitotic cells, lacking the portal triads or bile ducts (original magnification × 200). **(C)** Immunohistochemical showed β-catenin was positive on membrane, not nuclei (original magnification × 200). **(D)** The radiology images showed that the mass shrank significantly.

A 21-year-old male complained of asthenia and intermittent fever for 10 days was referred to our department for surgery from the department of hematology, as an ultrasound showed an undefined lesion on his liver. He has a history of severe aplastic anemia. Hepatitis B virus infection history or exposure in the infected area was denied. We learned that the patient took long-term exogenous testosterone for stimulating hematopoiesis. Upon examination he showed facial acne and facial hair. He was weak with a body-mass index of 20.5 kg/m^2^. On deep palpation he had increased mild tenderness of the right upper abdominal quadrant. His admission test was significant for declined leukocytes level (1.45 × 10^9^/L; lower limit of normal [LLN], 4.0 × 10^9^/L), declined erythrocytes level (1.17 × 10^12^/L; LLN, 4.0 × 10^12^/L) and declined platelets level (3 × 10^9^/L; LLN, 100 × 10^9^/L). Alpha fetoprotein (AFP) and carbohydrate antigen 19-9 were normal. His abdominal magnetic resonance imaging showed the lesion was markedly hyperintense at T2 with a range of 65.0 × 45.0 mm, and normal liver contour with no signs of liver cirrhosis. The mass was enhanced in the arterial phase and persisted in the portal venous and hepatobiliary phases (Figure 1A). Despite

the patient having asthenia and intermittent fever syndrome, the liver tuberculoma was a likely diagnosis. A test result for tuberculosis was negative. A liver biopsy was assigned, because the patient was scheduled for hematopoietic stem cell transplantation (HSCT) and immune inhibitor therapy. In this patient, liver biopsy revealed that the focal area of the liver plate was thickened with more pseudo-adenoid structures, slightly alien cells. Nevertheless, there was still divarication over the diagnosis of either benign or malignant as immunohistochemical stains for hepPar-1, glutamine synthetase, and heat shock protein 70 were positive, which are hepatocellular carcinoma markers (2). But cells were negative for glypican-3, and there was a lack of significant evidence for frank malignant behavior, Immunohistochemical staining showed that β-catenin was positive on the membranes, with < 10% positive for Ki67, consistent with a hepatic adenoma (Figures 1B,C). In the meantime, we excluded the extramedullary hematopoietic foci as no characteristic signs were found *via* microscopy. Extramedullary hematopoiesis, which commonly presents as granulocytes, erythrocytes, and giant cells are all visible, and erythroid hyperplasia is obvious (3).

The key to the correct diagnosis is recognizing that the presence of a liver mass in the background of normal liver parenchyma is unlikely to represent hepatocellular carcinoma, especially at a normal AFP level. Considering the patient's past medical history, negative blood tests, and the pathology of liver biopsy, the final diagnosis was identified as androgen-related hepatic adenoma. Then we withdrew the androgen for observation. Surprisingly, the lesion shrank significantly after 38 days. The patient was transferred to the department of hematology and completed hematopoietic stem cell transplantation. The lesion diminished gradually over a two-year follow-up (Figure 1D).

## Discussion

Gonadal hormones can induce adenoma described by Baum et al. first (4). Hepatic adenoma (HA) was subdivided into four types according to the Zucman-Rossi et al. (5) by using pathological type, and phenotype classification. They are inflammatory HA, steatotic HA, β-catenin activated HA, and unclassified HA. Androgen-related hepatic adenoma was characterized by some degree of histologic atypia or focal reticulin loss and majority being subtyped as β-catenin activated (6). And the images characteristic of β-catenin activated HA was diagnosed if the lesion was mainly heterogeneously hyper and hypointense, respectively, on T2 and T1 weighted with a central scar, and had no signal loss at chemical shift imaging. On contrast enhanced images, the lesion was enhanced in the arterial phase and showed persistence in either hepatobiliary phase. A steatotic HA was diagnosed if the lesion showed signal loss at T1 out-of-phase sequences compared with in-phase sequences. As for inflammatory HCA, this was diagnosed if the lesion was moderately to markedly hyperintense at T2 (7), with or without peripheral hyperintensity (pseudocapsule or atoll sign).

Androgen-related hepatic adenoma is not a malignant tumor. A rash choice of surgery will undoubtedly increase the incidence of intraoperative hemorrhage, postoperative infection, liver failure, and even death, and surgical intervention should be considered if there is sudden massive bleeding or malignant tendency (6). Non-negligibly, the androgen-receptor was found linked to the development of the hepatocellular carcinoma (8–10). Zucman-Rossi et al. reported hepatocellular carcinoma to be associated with adenoma or borderline lesions between carcinoma and adenoma, which can be found in 46% of the β-catenin-mutated tumors (5). More attention should be drawn to patients who take exogenous testosterone continuously. Careful monitoring with ultrasounds should be scheduled for them.

## Data Availability Statement

The original contributions presented in the study are included in the article/supplementary material, further inquiries can be directed to the corresponding author/s.

## Ethics Statement

The study was approved by the ethics committee of the Second Affiliated Hospital of Dalian Medical University. Written informed consent was obtained from the patient to have the case details and any accompanying images published.

## Author Contributions

JL, JS, ZG, JB, JY, and LW developed the main concept and designed the study. JL drafted the manuscript and JS edited it. DS literature search and data collection. KG assisted with the pathology part of the manuscript. LW supervised the whole process. All authors participated in the management of the patient in this case report.

## Conflict of Interest

The authors declare that the research was conducted in the absence of any commercial or financial relationships that could be construed as a potential conflict of interest.
